# Cactus Grandiflorus: A Study in Therapeutics

**Published:** 1894-10-27

**Authors:** 


### CACTUS GRANDIFLORUS—A STUDY IN THERAPEUTICS.

Cactus grandiflorus is a member of the large order, Cactaceæ, its natural habitat being tropical America. In that region medicinal virtues have long been attributed to it, and it has enjoyed a reputation as an anthelmintic, febrifuge, anti-neuralgic, and an anti-scorbutic. At the present time, however, whatever efficacy cactus may possess it is no longer highly esteemed in these respects, and is now principally used for its action on the heart. Every week there appears in one or other of the medical journals an advertisement of "cactina," a substance purporting to be the active principle of Cactus grandiflorus, isolated by Sultan. This advertisement boldly states that cactina is valuable in all diseases of the heart, but, of course, such a statement must be accepted with due reserve. In the following lines an endeavour is made to point out the conditions in which the drug seems likely, from the experience of competent observers, to be really of service.

Gregory, of California, from an experience of twenty years, considers a tincture of the drug serviceable in those cases in which, dilatation having outstripped hypertrophy of the heart muscle, digitalis is no longer efficacious. It is the only remedy which he uses in functional valvular disease. In the same strain Engstad writes,[1] "I now depend upon it in preference to digitalis in functional disorders," and records its successful employment in cases of failing heart after typhoid fever, when digitalis and strychnine had both been tried without avail. Again, Aulde writes[2] that he considers it most useful in regulating the cardiac movements, either in functional or valvular disorders. He supports this conclusion by records of its beneficial action in angina pectoris, organic lesions, feeble and irritable heart as a consequence of excessive sexual indulgence, the morphia habit, tobacco poisoning, menorrhagia, &c. It appeared to him to be a safer remedy than digitalis because it was free from cumulative action.

Orlando Jones[3] found cactus useful in cases where, on account of excessive feebleness of the heart muscle, digitalis was contra-indicated. The drug strengthened the contractions, increased the vascular tension, and accelerated the circulation, its therapeutic effect being marked in all asthenic conditions of the heart, whatever their origin.

Huchard and O'Meara have also found cactus useful in organic heart affections. In aortic regurgitation, when hypertrophy was insufficient for complete compensation, digitalis being contra-indicated because of its effect in prolonging the diastolic period, they used cactus with success, the diastole being shortened while the systole was prolonged. Huchard found also that in mitral regurgitation with dilatation of the ventricle, cactus occasionally gave better results than digitalis.

The experience of Williams, Harvey, Bird, and others, confirms the above, and these observers give cactus with benefit in cases of acute and sub-acute rheumatism, the drug rendering cardiac complications less liable to occur. They agree with the views of Pitzer, who thinks that cactus acts upon the cardiac plexus, improving the nutrition of the organ.

One of the latest writers on the merits of cactus, is,

Gibelli.[4] He found that after the use of cactus the pulse wave was increased both in height and force, the increase being due, not to increase of blood pressure, but to a diminished tension in the vessels, as shown by a marked dicrotism in the sphygmographic tracings. Such a dilating effect on the vessels might explain the good effects obtained by other observers in angina pectoris. In most cases of arythmia, the drug restored the regularity of the heart beat without increasing its frequency. It had also a quieting effect on the nervous system, for in several cases dyspnœa was relieved, while it also considerably diminished the pains of rheumatism and neuralgia.

The action of "cactina" appears to be almost identical with that of an alcoholic extract of cactus. Myers[5], from experiments made on dogs, concluded that cactina increased the heart's energy, rendering its beats quicker and stronger, through an action on the accelerator ganglia. Through the increase of cardiac force and excitation of the vasamotor centre, it produced a rise of blood pressure with increased force of the pulse. Again, acting on the nervous system it produced excitation of the motor centres of the cord, causing exaggerated reflexes. Cactina, therefore, appeared to have an action resembling that of strychnine, and as it caused shortening of the period of diastole, it was tried by Myers in cases of valvular disease other than mitral stenosis, with much success. In tobacco-heart, also, and in other functional disorders, he found cactina as efficacious as the extract of the plant.

R. W. Wilcox also used cactina as well as the extract, but he preferred the latter, as he found that cactina was in some cases apt to produce too great a rise of blood pressure, leading to hæmorrhages. H. C. Wood came to a similar conclusion, and preferred to employ the aqueous extract, which in his hands yielded excellent results.

To summarize, there are two preparations in the market which may be used with safety and advantage, the tincture and the fluid extract. The dose of either of these varies, according to the reports above alluded to, between five and ten or fifteen drops. The extract is given three times daily, but the tincture is employed every two or three hours.

In its action cactus is a cardiac tonic, unobjectionable to the palate, and free from cumulative effect. It has no narcotic action, and produces no material constriction of the arterioles like digitalis. Perhaps (Gibelli) it has even an opposite effect. It acts as a valuable regulator of the heart, whether the irregularity be the result of functional disorder or of valvular lesions.

In cases of palpitation accompanying uterine disorders, or the result of an enfeebled condition of the digestive system, cactus has yielded brilliant results. In females the latter condition is often accompanied with menorrhagia or metrorrhagia, and in such cases the treatment with cactus, in order to bring about a radical cure, must be conjoined with other measures directed to the stomach and uterus.

Cactus has proved serviceable also in cases of dilatation with hypertrophy, accompanied with a murmur, an indication that dilatation has outstripped hypertrophy. It may succeed in the case of dilatation with anasarca, with or without valvular disease, even when digitalis, cathartics, and diuretics have failed.

The irregular and enfeebled heart resulting from the morphia or chloral habit appears to be greatly benefited by a course of cactus, and the same may be said of the irregularities resulting from excess in tea, tobacco, or sexual indulgence. Of course in such cases any treatment must also involve the removal of the initial cause.

In mitral stenosis digitalis is to be preferred, from the fact that it prolongs the diastolic period, and thus gives greater time for the ventricle to fill, but in aortic incompetence the short diastole induced by cactus should be of great benefit.

Before failure is accredited to the drug in any case one must be quite sure that the preparation used is an active one, for there are many useless ones in the market. The writer of these lines has found that the preparations of either Merck or Parke, Davis, and Co. are to be relied upon, but there may be many others equally good.

[1] Therapeutic Gazette, Sept., 1890. [2] Therap. Gaz., May, 1891. [3] Brit. Med. Jour., Jan., 1890. [4] Gazz. degli Ospitali, May, 1893. [5] New York Medical Journal, June, 1891.

